# Bis[2,3,4-trimethyl-5-[(3,4,5-trimethyl-2*H*-pyrrol-2-yl­idene-κ*N*)meth­yl]-1*H*-pyrrolato-κ*N*]copper(II)^1^

**DOI:** 10.1107/S1600536812040998

**Published:** 2012-10-06

**Authors:** Sultan S. Erdem, Frank R. Fronczek, Steven F. Watkins

**Affiliations:** a Department of Chemistry, Louisiana State University, Baton Rouge LA 70803-1804 USA

## Abstract

In the title complex, [Cu(C_15_H_19_N_2_)_2_] or [Cu(*L*_2_)] (H*L* is 3,3′,4,4′,5,5′-hexa­methyl­pyrromethene), the Cu^II^ atom is coordinated by four N atoms [Cu—N 1.939 (2)–1.976 (2) Å] from two *L* ligands in a distorted tetra­hedral geometry. The mean planes of the CuN_2_C_3_ metallocyclic rings form a dihedral angle of 72.73 (6)°. In the *L* ligands, the pyrrole rings are inclined to each other at dihedral angles of 3.03 (7) and 9.83 (7)°. The crystal packing exhibits weak inter­molecular C—H⋯π inter­actions, which form chains in [100].

## Related literature

For the structure of the neutral ligand, see: Mroginski *et al.* (2005 ▶). For the structures of related organometallic complexes, see: Elder & Penfold (1969 ▶); Cotton *et al.* (1970 ▶); Fergusson *et al.* (1971 ▶). For a description of the Cambridge Structural Database, see: Allen (2002 ▶). For transition metal complexes of dipyrromethenes, see: Bruckner *et al.* (1997 ▶); Zhang *et al.* (1998 ▶). For the chemistry and applications of pyrrole derivatives, see: Dolphin (1979 ▶); Falk (1989 ▶). For the synthesis of the title compound, see: Murakami & Sakata (1968 ▶). For *IDEAL* software, see: Gould *et al.* (1988 ▶).
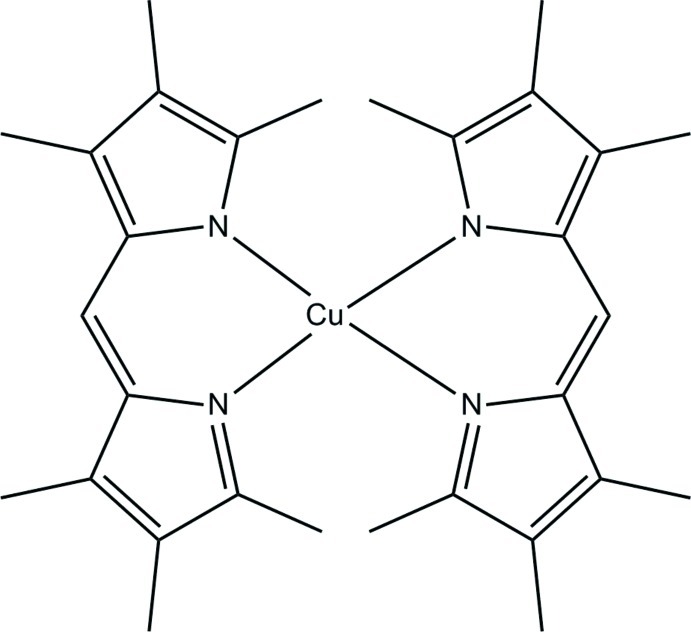


## Experimental

### 

#### Crystal data

[Cu(C_15_H_19_N_2_)_2_]
*M* *_r_* = 518.18
Triclinic, 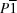

*a* = 7.9737 (1) Å
*b* = 12.0896 (3) Å
*c* = 13.9411 (4) Å
α = 92.8065 (8)°
β = 105.4205 (8)°
γ = 91.9772 (18)°
*V* = 1292.39 (5) Å^3^
*Z* = 2
Mo *K*α radiation
μ = 0.87 mm^−1^
*T* = 120 K
0.18 × 0.10 × 0.02 mm

#### Data collection

Nonius KappaCCD diffractometer
Absorption correction: multi-scan (*HKL* *SCALEPACK*; Otwinowski & Minor, 1997 ▶) *T*_min_ = 0.859, *T*_max_ = 0.983
20968 measured reflections
7342 independent reflections
5581 reflections with *I* > 2σ(*I*)
*R*_int_ = 0.052

#### Refinement

*R*[*F* ^2^ > 2σ(*F* ^2^)] = 0.050
*wR*(*F* ^2^) = 0.117
*S* = 1.02
7342 reflections
328 parameters
H-atom parameters constrained
Δρ_max_ = 0.49 e Å^−3^
Δρ_min_ = −0.48 e Å^−3^

### 

Data collection: *COLLECT* (Nonius, 2000 ▶); cell refinement: *DENZO* and *SCALEPACK* (Otwinowski & Minor, 1997 ▶); data reduction: *DENZO* and *SCALEPACK*; program(s) used to solve structure: *SIR2002* (Burla *et al.*, 2003 ▶); program(s) used to refine structure: *SHELXL97* (Sheldrick, 2008 ▶); molecular graphics: *ORTEP-3 for Windows* (Farrugia, 1997 ▶); software used to prepare material for publication: *WinGX* publication routines (Farrugia, 1999 ▶).

## Supplementary Material

Crystal structure: contains datablock(s) global, I. DOI: 10.1107/S1600536812040998/cv5335sup1.cif

Structure factors: contains datablock(s) I. DOI: 10.1107/S1600536812040998/cv5335Isup2.hkl

Additional supplementary materials:  crystallographic information; 3D view; checkCIF report

## Figures and Tables

**Table 1:** Hydrogen-bond geometry (Å, °) *Cg* is the centroid of the N3/C16–C19 pyrrole ring.

*D*—H⋯*A*	*D*—H	H⋯*A*	*D*⋯*A*	*D*—H⋯*A*
C29—H29*C*⋯*Cg* ^i^	0.98	2.78	3.551 (3)	136

Symmetry code: (i) 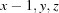.

**Fig. 1.**
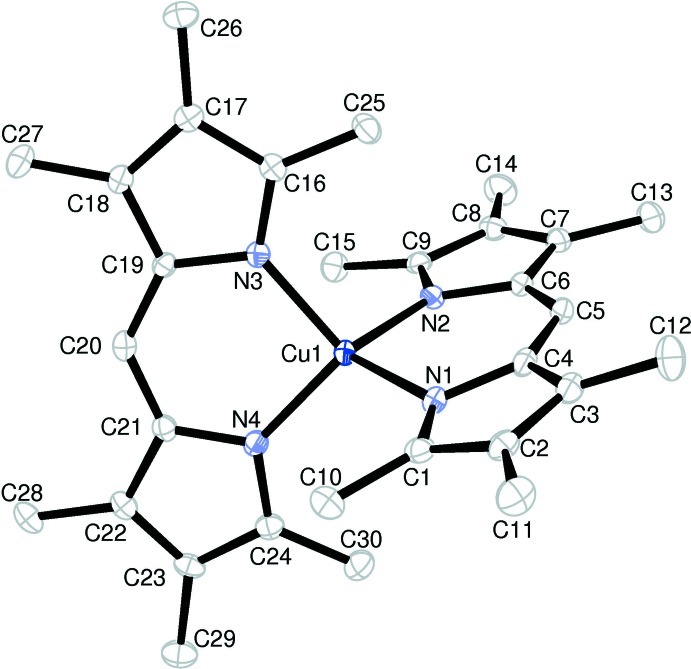
View of (I) (50% probability displacement ellipsoids). H atoms are not shown.

## supplementary crystallographic information

### Comment

Dipyrromethenes are fully conjugated anionic ligands which create stable transition metal complexes (Bruckner *et al.*, 1997, Zhang *et al.*, 1998). The high chemical stability of the title compound (I) is associated with its extended aromatic structure. Pyrroles also form crucial building blocks for bile pigments, linear polypyrroles and porphyrins, and have been investigated for treatment of cancer by photodynamic therapy (Dolphin, 1979; Falk, 1989).

The structure of the neutral protonated ligand, C_15_H_20_N_2_, has been determined (Mroginski *et al.*, 2005, CCDC refcode PALFEO, Allen, 2002). Comparison of the ligated anions in I with the neutral species shows excellent structural coincidence for all non-hydrogen atoms, with δ_r.m.s._ = 0.091 Å (IDEAL, Gould *et al.*, 1988).

Intermolecular interactions include weak C—H···π contacts involving methyl group C15 and the pyrrole ring N3/C16-C19 (Table 1), thus forming chains in the [100] direction.

### Experimental

The title compound was synthesized by heating a suspension of dipyrromethene hydrochloride, copper acetate monohydrate and sodium acetate in ethanol-water (Murakami & Sakata, 1968).

### Refinement

H atoms were placed in calculated positions, guided by difference maps, with C—H bond distances 0.95–0.98 Å, *U*_iso_ = 1.2*U*_eq_ of the attached carbon atom (1.5 for methyl), and thereafter treated as riding. A torsional parameter was refined for each methyl group.

### Crystal data

**Table:**

[Cu(C_15_H_19_N_2_)_2_]	*Z* = 2
*M**_r_* = 518.18	*F*(000) = 550
Triclinic, *P*1	*D*_x_ = 1.331 Mg m^−^^3^
Hall symbol: -P 1	Mo *K*α radiation, λ = 0.71073 Å
*a* = 7.9737 (1) Å	Cell parameters from 6313 reflections
*b* = 12.0896 (3) Å	θ = 2.5–30°
*c* = 13.9411 (4) Å	µ = 0.87 mm^−^^1^
α = 92.8065 (8)°	*T* = 120 K
β = 105.4205 (8)°	Lath fragment, metallic green
γ = 91.9772 (18)°	0.18 × 0.10 × 0.02 mm
*V* = 1292.39 (5) Å^3^	

### Data collection

**Table:**

Nonius KappaCCD diffractometer	7342 independent reflections
Radiation source: sealed tube	5581 reflections with *I* > 2σ(*I*)
Horizonally mounted graphite crystal monochromator	*R*_int_ = 0.052
Detector resolution: 9 pixels mm^-1^	θ_max_ = 30.0°, θ_min_ = 2.6°
φ and ω scans	*h* = −11→10
Absorption correction: multi-scan (*HKL**SCALEPACK*; Otwinowski & Minor, 1997)	*k* = −17→16
*T*_min_ = 0.859, *T*_max_ = 0.983	*l* = 0→19
20968 measured reflections	

### Refinement

**Table:**

Refinement on *F*^2^	Primary atom site location: structure-invariant direct methods
Least-squares matrix: full	Secondary atom site location: difference Fourier map
*R*[*F*^2^ > 2σ(*F*^2^)] = 0.050	Hydrogen site location: inferred from neighbouring sites
*wR*(*F*^2^) = 0.117	H-atom parameters constrained
*S* = 1.02	*w* = 1/[σ^2^(*F*_o_^2^) + (0.0459*P*)^2^ + 1.301*P*] where *P* = (*F*_o_^2^ + 2*F*_c_^2^)/3
7342 reflections	(Δ/σ)_max_ = 0.001
328 parameters	Δρ_max_ = 0.49 e Å^−^^3^
0 restraints	Δρ_min_ = −0.48 e Å^−^^3^
0 constraints	

### Special details

**Table:**

Geometry. All e.s.d.'s (except the e.s.d. in the dihedral angle between two l.s. planes) are estimated using the full covariance matrix. The cell e.s.d.'s are taken into account individually in the estimation of e.s.d.'s in distances, angles and torsion angles; correlations between e.s.d.'s in cell parameters are only used when they are defined by crystal symmetry. An approximate (isotropic) treatment of cell e.s.d.'s is used for estimating e.s.d.'s involving l.s. planes.

### Fractional atomic coordinates and isotropic or equivalent isotropic displacement parameters (Å^2^)

**Table:**

	*x*	*y*	*z*	*U*_iso_*/*U*_eq_	
C1	0.0551 (3)	0.30099 (19)	0.06874 (18)	0.0170 (4)	
C2	0.1508 (3)	0.3865 (2)	0.03636 (18)	0.0182 (5)	
C3	0.2301 (3)	0.45455 (19)	0.11937 (18)	0.0177 (5)	
C4	0.1808 (3)	0.41018 (18)	0.20159 (18)	0.0163 (4)	
C5	0.2270 (3)	0.45235 (19)	0.30043 (18)	0.0166 (4)	
H5	0.293	0.521	0.313	0.02*	
C6	0.1901 (3)	0.40808 (18)	0.38310 (18)	0.0161 (4)	
C7	0.2300 (3)	0.45367 (19)	0.48376 (18)	0.0182 (5)	
C8	0.1726 (3)	0.3752 (2)	0.53829 (18)	0.0180 (5)	
C9	0.0982 (3)	0.28389 (19)	0.47050 (18)	0.0167 (4)	
C10	−0.0506 (3)	0.2066 (2)	0.00526 (19)	0.0228 (5)	
H10A	−0.0581	0.1444	0.0467	0.034*	
H10B	0.005	0.1832	−0.0467	0.034*	
H10C	−0.168	0.2302	−0.0259	0.034*	
C11	0.1629 (3)	0.3977 (2)	−0.06840 (19)	0.0246 (5)	
H11A	0.1608	0.4762	−0.0831	0.037*	
H11B	0.064	0.3566	−0.115	0.037*	
H11C	0.2719	0.3677	−0.0755	0.037*	
C12	0.3503 (4)	0.5533 (2)	0.1225 (2)	0.0278 (6)	
H12A	0.4591	0.5289	0.1102	0.042*	
H12B	0.3756	0.5928	0.1882	0.042*	
H12C	0.295	0.603	0.0711	0.042*	
C13	0.3121 (3)	0.5663 (2)	0.5217 (2)	0.0239 (5)	
H13A	0.221	0.6179	0.5241	0.036*	
H13B	0.3791	0.5927	0.477	0.036*	
H13C	0.39	0.5619	0.5887	0.036*	
C14	0.1815 (3)	0.3850 (2)	0.64745 (19)	0.0273 (6)	
H14A	0.2834	0.4324	0.683	0.041*	
H14B	0.1911	0.3113	0.674	0.041*	
H14C	0.0756	0.4179	0.6565	0.041*	
C15	0.0127 (3)	0.1785 (2)	0.49176 (19)	0.0206 (5)	
H15A	−0.1134	0.1864	0.4772	0.031*	
H15B	0.0591	0.1637	0.5621	0.031*	
H15C	0.0365	0.1169	0.4498	0.031*	
C16	0.3462 (3)	0.07330 (19)	0.34173 (17)	0.0149 (4)	
C17	0.4012 (3)	−0.03756 (19)	0.35150 (16)	0.0155 (4)	
C18	0.2537 (3)	−0.10652 (19)	0.31410 (17)	0.0152 (4)	
C19	0.1112 (3)	−0.03552 (18)	0.28089 (16)	0.0133 (4)	
C20	−0.0604 (3)	−0.06651 (18)	0.23318 (16)	0.0146 (4)	
H20	−0.0881	−0.1441	0.225	0.018*	
C21	−0.1988 (3)	0.00086 (18)	0.19539 (17)	0.0150 (4)	
C22	−0.3776 (3)	−0.03267 (19)	0.15404 (17)	0.0161 (4)	
C23	−0.4672 (3)	0.0633 (2)	0.13321 (17)	0.0169 (4)	
C24	−0.3418 (3)	0.15334 (19)	0.16063 (17)	0.0169 (4)	
C25	0.4632 (3)	0.1757 (2)	0.37120 (19)	0.0205 (5)	
H25A	0.4076	0.2376	0.3343	0.031*	
H25B	0.5741	0.1632	0.3557	0.031*	
H25C	0.4844	0.1934	0.4429	0.031*	
C26	0.5858 (3)	−0.0683 (2)	0.39435 (18)	0.0202 (5)	
H26A	0.5903	−0.1491	0.3948	0.03*	
H26B	0.6288	−0.0357	0.4627	0.03*	
H26C	0.6588	−0.04	0.3536	0.03*	
C27	0.2415 (3)	−0.23026 (19)	0.3073 (2)	0.0211 (5)	
H27A	0.3589	−0.2584	0.3252	0.032*	
H27B	0.1806	−0.257	0.239	0.032*	
H27C	0.1769	−0.2566	0.3532	0.032*	
C28	−0.4551 (3)	−0.1488 (2)	0.13782 (19)	0.0221 (5)	
H28A	−0.5556	−0.1535	0.1656	0.033*	
H28B	−0.3677	−0.1995	0.171	0.033*	
H28C	−0.4927	−0.1695	0.0662	0.033*	
C29	−0.6605 (3)	0.0712 (2)	0.09145 (19)	0.0234 (5)	
H29A	−0.6925	0.0522	0.0195	0.035*	
H29B	−0.693	0.147	0.1037	0.035*	
H29C	−0.7221	0.0196	0.1239	0.035*	
C30	−0.3737 (3)	0.2739 (2)	0.1535 (2)	0.0235 (5)	
H30A	−0.3121	0.314	0.2165	0.035*	
H30B	−0.4989	0.2846	0.1402	0.035*	
H30C	−0.331	0.3023	0.0993	0.035*	
N1	0.0723 (2)	0.31450 (16)	0.16751 (15)	0.0158 (4)	
N2	0.1078 (2)	0.30305 (15)	0.37825 (14)	0.0150 (4)	
N3	0.1743 (2)	0.07482 (15)	0.30066 (14)	0.0141 (4)	
N4	−0.1810 (2)	0.11631 (16)	0.19835 (15)	0.0154 (4)	
Cu1	0.03402 (3)	0.20344 (2)	0.25992 (2)	0.01496 (9)	

### Atomic displacement parameters (Å^2^)

**Table:**

	*U*^11^	*U*^22^	*U*^33^	*U*^12^	*U*^13^	*U*^23^
C1	0.0148 (10)	0.0179 (11)	0.0179 (11)	0.0046 (8)	0.0028 (8)	0.0022 (9)
C2	0.0173 (11)	0.0181 (11)	0.0208 (12)	0.0059 (8)	0.0071 (9)	0.0030 (9)
C3	0.0178 (11)	0.0155 (11)	0.0209 (12)	0.0026 (8)	0.0063 (9)	0.0036 (9)
C4	0.0165 (11)	0.0118 (10)	0.0217 (12)	0.0026 (8)	0.0064 (8)	0.0034 (9)
C5	0.0148 (10)	0.0116 (10)	0.0223 (12)	0.0016 (8)	0.0028 (8)	0.0012 (9)
C6	0.0146 (10)	0.0118 (10)	0.0202 (12)	0.0015 (8)	0.0019 (8)	0.0008 (9)
C7	0.0151 (11)	0.0169 (11)	0.0208 (12)	0.0030 (8)	0.0018 (8)	−0.0012 (9)
C8	0.0159 (11)	0.0203 (12)	0.0166 (11)	0.0027 (8)	0.0026 (8)	−0.0009 (9)
C9	0.0126 (10)	0.0179 (11)	0.0193 (12)	0.0041 (8)	0.0027 (8)	0.0033 (9)
C10	0.0208 (12)	0.0254 (13)	0.0203 (13)	−0.0014 (9)	0.0030 (9)	−0.0015 (10)
C11	0.0264 (13)	0.0280 (14)	0.0229 (13)	0.0025 (10)	0.0117 (10)	0.0067 (11)
C12	0.0325 (14)	0.0204 (13)	0.0333 (15)	−0.0065 (10)	0.0147 (11)	0.0030 (11)
C13	0.0266 (13)	0.0182 (12)	0.0233 (13)	−0.0004 (9)	0.0016 (10)	−0.0059 (10)
C14	0.0287 (14)	0.0321 (15)	0.0194 (13)	−0.0012 (11)	0.0044 (10)	−0.0015 (11)
C15	0.0218 (12)	0.0202 (12)	0.0211 (12)	0.0001 (9)	0.0080 (9)	0.0025 (10)
C16	0.0157 (10)	0.0170 (11)	0.0126 (10)	0.0000 (8)	0.0053 (8)	−0.0003 (8)
C17	0.0187 (11)	0.0186 (11)	0.0104 (10)	0.0029 (8)	0.0057 (8)	0.0017 (8)
C18	0.0193 (11)	0.0141 (10)	0.0132 (11)	0.0019 (8)	0.0058 (8)	0.0016 (8)
C19	0.0150 (10)	0.0126 (10)	0.0126 (10)	0.0011 (8)	0.0042 (8)	0.0015 (8)
C20	0.0193 (11)	0.0133 (10)	0.0131 (10)	−0.0014 (8)	0.0080 (8)	0.0003 (8)
C21	0.0173 (11)	0.0150 (11)	0.0128 (11)	−0.0013 (8)	0.0044 (8)	0.0005 (8)
C22	0.0165 (11)	0.0197 (11)	0.0126 (11)	−0.0027 (8)	0.0057 (8)	−0.0017 (9)
C23	0.0139 (11)	0.0246 (12)	0.0120 (11)	0.0003 (8)	0.0034 (8)	−0.0001 (9)
C24	0.0172 (11)	0.0191 (11)	0.0146 (11)	0.0030 (8)	0.0042 (8)	0.0014 (9)
C25	0.0164 (11)	0.0206 (12)	0.0235 (13)	−0.0031 (9)	0.0048 (9)	−0.0015 (10)
C26	0.0183 (11)	0.0258 (13)	0.0166 (12)	0.0058 (9)	0.0039 (9)	0.0028 (10)
C27	0.0240 (12)	0.0149 (11)	0.0262 (13)	0.0051 (9)	0.0091 (10)	0.0028 (10)
C28	0.0174 (11)	0.0235 (13)	0.0235 (13)	−0.0063 (9)	0.0037 (9)	−0.0030 (10)
C29	0.0168 (12)	0.0332 (14)	0.0200 (12)	0.0023 (10)	0.0046 (9)	0.0010 (11)
C30	0.0227 (12)	0.0205 (12)	0.0270 (14)	0.0047 (9)	0.0054 (10)	0.0030 (10)
N1	0.0166 (9)	0.0128 (9)	0.0174 (10)	0.0012 (7)	0.0032 (7)	0.0025 (7)
N2	0.0142 (9)	0.0142 (9)	0.0155 (10)	0.0008 (7)	0.0024 (7)	0.0001 (7)
N3	0.0151 (9)	0.0130 (9)	0.0145 (9)	0.0002 (7)	0.0045 (7)	0.0005 (7)
N4	0.0144 (9)	0.0139 (9)	0.0175 (10)	0.0009 (7)	0.0035 (7)	0.0018 (7)
Cu1	0.01634 (14)	0.01081 (13)	0.01597 (15)	−0.00039 (9)	0.00133 (10)	0.00120 (10)

### Geometric parameters (Å, º)

**Table:**

C1—N1	1.348 (3)	C16—C25	1.496 (3)
C1—C2	1.425 (3)	C17—C18	1.384 (3)
C1—C10	1.495 (3)	C17—C26	1.501 (3)
C2—C3	1.378 (3)	C18—C19	1.438 (3)
C2—C11	1.501 (4)	C18—C27	1.493 (3)
C3—C4	1.429 (3)	C19—C20	1.383 (3)
C3—C12	1.497 (3)	C19—N3	1.397 (3)
C4—C5	1.394 (3)	C20—C21	1.397 (3)
C4—N1	1.404 (3)	C20—H20	0.95
C5—C6	1.390 (3)	C21—N4	1.395 (3)
C5—H5	0.95	C21—C22	1.425 (3)
C6—N2	1.399 (3)	C22—C23	1.386 (3)
C6—C7	1.431 (3)	C22—C28	1.496 (3)
C7—C8	1.384 (3)	C23—C24	1.418 (3)
C7—C13	1.499 (3)	C23—C29	1.504 (3)
C8—C9	1.420 (3)	C24—N4	1.351 (3)
C8—C14	1.503 (4)	C24—C30	1.492 (3)
C9—N2	1.339 (3)	C25—H25A	0.98
C9—C15	1.503 (3)	C25—H25B	0.98
C10—H10A	0.98	C25—H25C	0.98
C10—H10B	0.98	C26—H26A	0.98
C10—H10C	0.98	C26—H26B	0.98
C11—H11A	0.98	C26—H26C	0.98
C11—H11B	0.98	C27—H27A	0.98
C11—H11C	0.98	C27—H27B	0.98
C12—H12A	0.98	C27—H27C	0.98
C12—H12B	0.98	C28—H28A	0.98
C12—H12C	0.98	C28—H28B	0.98
C13—H13A	0.98	C28—H28C	0.98
C13—H13B	0.98	C29—H29A	0.98
C13—H13C	0.98	C29—H29B	0.98
C14—H14A	0.98	C29—H29C	0.98
C14—H14B	0.98	C30—H30A	0.98
C14—H14C	0.98	C30—H30B	0.98
C15—H15A	0.98	C30—H30C	0.98
C15—H15B	0.98	N1—Cu1	1.9762 (19)
C15—H15C	0.98	N2—Cu1	1.9385 (19)
C16—N3	1.339 (3)	N3—Cu1	1.9650 (19)
C16—C17	1.426 (3)	N4—Cu1	1.9471 (19)
			
N1—C1—C2	110.9 (2)	C20—C19—N3	123.4 (2)
N1—C1—C10	122.6 (2)	C20—C19—C18	127.7 (2)
C2—C1—C10	126.5 (2)	N3—C19—C18	108.78 (18)
C3—C2—C1	106.8 (2)	C19—C20—C21	128.7 (2)
C3—C2—C11	127.4 (2)	C19—C20—H20	115.6
C1—C2—C11	125.8 (2)	C21—C20—H20	115.6
C2—C3—C4	106.9 (2)	N4—C21—C20	123.4 (2)
C2—C3—C12	126.1 (2)	N4—C21—C22	108.86 (19)
C4—C3—C12	127.0 (2)	C20—C21—C22	127.6 (2)
C5—C4—N1	123.5 (2)	C23—C22—C21	106.8 (2)
C5—C4—C3	127.6 (2)	C23—C22—C28	126.4 (2)
N1—C4—C3	108.9 (2)	C21—C22—C28	126.8 (2)
C6—C5—C4	129.1 (2)	C22—C23—C24	106.8 (2)
C6—C5—H5	115.5	C22—C23—C29	126.8 (2)
C4—C5—H5	115.5	C24—C23—C29	126.4 (2)
C5—C6—N2	122.2 (2)	N4—C24—C23	110.6 (2)
C5—C6—C7	129.2 (2)	N4—C24—C30	122.1 (2)
N2—C6—C7	108.6 (2)	C23—C24—C30	127.3 (2)
C8—C7—C6	106.7 (2)	C16—C25—H25A	109.5
C8—C7—C13	126.8 (2)	C16—C25—H25B	109.5
C6—C7—C13	126.5 (2)	H25A—C25—H25B	109.5
C7—C8—C9	106.7 (2)	C16—C25—H25C	109.5
C7—C8—C14	127.0 (2)	H25A—C25—H25C	109.5
C9—C8—C14	126.2 (2)	H25B—C25—H25C	109.5
N2—C9—C8	110.9 (2)	C17—C26—H26A	109.5
N2—C9—C15	121.2 (2)	C17—C26—H26B	109.5
C8—C9—C15	127.9 (2)	H26A—C26—H26B	109.5
C1—C10—H10A	109.5	C17—C26—H26C	109.5
C1—C10—H10B	109.5	H26A—C26—H26C	109.5
H10A—C10—H10B	109.5	H26B—C26—H26C	109.5
C1—C10—H10C	109.5	C18—C27—H27A	109.5
H10A—C10—H10C	109.5	C18—C27—H27B	109.5
H10B—C10—H10C	109.5	H27A—C27—H27B	109.5
C2—C11—H11A	109.5	C18—C27—H27C	109.5
C2—C11—H11B	109.5	H27A—C27—H27C	109.5
H11A—C11—H11B	109.5	H27B—C27—H27C	109.5
C2—C11—H11C	109.5	C22—C28—H28A	109.5
H11A—C11—H11C	109.5	C22—C28—H28B	109.5
H11B—C11—H11C	109.5	H28A—C28—H28B	109.5
C3—C12—H12A	109.5	C22—C28—H28C	109.5
C3—C12—H12B	109.5	H28A—C28—H28C	109.5
H12A—C12—H12B	109.5	H28B—C28—H28C	109.5
C3—C12—H12C	109.5	C23—C29—H29A	109.5
H12A—C12—H12C	109.5	C23—C29—H29B	109.5
H12B—C12—H12C	109.5	H29A—C29—H29B	109.5
C7—C13—H13A	109.5	C23—C29—H29C	109.5
C7—C13—H13B	109.5	H29A—C29—H29C	109.5
H13A—C13—H13B	109.5	H29B—C29—H29C	109.5
C7—C13—H13C	109.5	C24—C30—H30A	109.5
H13A—C13—H13C	109.5	C24—C30—H30B	109.5
H13B—C13—H13C	109.5	H30A—C30—H30B	109.5
C8—C14—H14A	109.5	C24—C30—H30C	109.5
C8—C14—H14B	109.5	H30A—C30—H30C	109.5
H14A—C14—H14B	109.5	H30B—C30—H30C	109.5
C8—C14—H14C	109.5	C1—N1—C4	106.43 (19)
H14A—C14—H14C	109.5	C1—N1—Cu1	128.71 (16)
H14B—C14—H14C	109.5	C4—N1—Cu1	121.56 (15)
C9—C15—H15A	109.5	C9—N2—C6	107.10 (19)
C9—C15—H15B	109.5	C9—N2—Cu1	127.50 (16)
H15A—C15—H15B	109.5	C6—N2—Cu1	125.33 (16)
C9—C15—H15C	109.5	C16—N3—C19	106.97 (18)
H15A—C15—H15C	109.5	C16—N3—Cu1	128.25 (15)
H15B—C15—H15C	109.5	C19—N3—Cu1	124.45 (14)
N3—C16—C17	111.2 (2)	C24—N4—C21	106.98 (18)
N3—C16—C25	123.5 (2)	C24—N4—Cu1	127.95 (16)
C17—C16—C25	125.3 (2)	C21—N4—Cu1	124.74 (15)
C18—C17—C16	106.57 (19)	N2—Cu1—N4	133.10 (8)
C18—C17—C26	128.8 (2)	N2—Cu1—N3	101.81 (8)
C16—C17—C26	124.7 (2)	N4—Cu1—N3	95.11 (8)
C17—C18—C19	106.50 (19)	N2—Cu1—N1	95.00 (8)
C17—C18—C27	127.7 (2)	N4—Cu1—N1	109.25 (8)
C19—C18—C27	125.8 (2)	N3—Cu1—N1	126.54 (8)
			
N1—C1—C2—C3	0.3 (3)	C2—C1—N1—C4	0.0 (2)
C10—C1—C2—C3	−179.1 (2)	C10—C1—N1—C4	179.4 (2)
N1—C1—C2—C11	179.5 (2)	C2—C1—N1—Cu1	−159.31 (16)
C10—C1—C2—C11	0.2 (4)	C10—C1—N1—Cu1	20.1 (3)
C1—C2—C3—C4	−0.4 (2)	C5—C4—N1—C1	179.0 (2)
C11—C2—C3—C4	−179.7 (2)	C3—C4—N1—C1	−0.3 (2)
C1—C2—C3—C12	177.4 (2)	C5—C4—N1—Cu1	−19.9 (3)
C11—C2—C3—C12	−1.8 (4)	C3—C4—N1—Cu1	160.86 (15)
C2—C3—C4—C5	−178.8 (2)	C8—C9—N2—C6	0.2 (2)
C12—C3—C4—C5	3.4 (4)	C15—C9—N2—C6	178.6 (2)
C2—C3—C4—N1	0.4 (3)	C8—C9—N2—Cu1	177.39 (15)
C12—C3—C4—N1	−177.4 (2)	C15—C9—N2—Cu1	−4.3 (3)
N1—C4—C5—C6	5.1 (4)	C5—C6—N2—C9	177.6 (2)
C3—C4—C5—C6	−175.8 (2)	C7—C6—N2—C9	−0.5 (2)
C4—C5—C6—N2	5.8 (4)	C5—C6—N2—Cu1	0.3 (3)
C4—C5—C6—C7	−176.6 (2)	C7—C6—N2—Cu1	−177.68 (14)
C5—C6—C7—C8	−177.3 (2)	C17—C16—N3—C19	−0.8 (3)
N2—C6—C7—C8	0.5 (2)	C25—C16—N3—C19	178.2 (2)
C5—C6—C7—C13	4.7 (4)	C17—C16—N3—Cu1	−174.31 (15)
N2—C6—C7—C13	−177.5 (2)	C25—C16—N3—Cu1	4.6 (3)
C6—C7—C8—C9	−0.3 (2)	C20—C19—N3—C16	−176.4 (2)
C13—C7—C8—C9	177.6 (2)	C18—C19—N3—C16	1.2 (2)
C6—C7—C8—C14	−178.6 (2)	C20—C19—N3—Cu1	−2.5 (3)
C13—C7—C8—C14	−0.6 (4)	C18—C19—N3—Cu1	175.09 (15)
C7—C8—C9—N2	0.1 (3)	C23—C24—N4—C21	0.5 (3)
C14—C8—C9—N2	178.3 (2)	C30—C24—N4—C21	179.0 (2)
C7—C8—C9—C15	−178.1 (2)	C23—C24—N4—Cu1	−173.08 (16)
C14—C8—C9—C15	0.2 (4)	C30—C24—N4—Cu1	5.5 (3)
N3—C16—C17—C18	0.0 (3)	C20—C21—N4—C24	−176.6 (2)
C25—C16—C17—C18	−178.9 (2)	C22—C21—N4—C24	0.1 (3)
N3—C16—C17—C26	179.8 (2)	C20—C21—N4—Cu1	−2.8 (3)
C25—C16—C17—C26	0.8 (4)	C22—C21—N4—Cu1	173.90 (15)
C16—C17—C18—C19	0.8 (2)	C9—N2—Cu1—N4	50.3 (2)
C26—C17—C18—C19	−179.0 (2)	C6—N2—Cu1—N4	−133.06 (17)
C16—C17—C18—C27	179.9 (2)	C9—N2—Cu1—N3	−58.40 (19)
C26—C17—C18—C27	0.2 (4)	C6—N2—Cu1—N3	118.25 (18)
C17—C18—C19—C20	176.2 (2)	C9—N2—Cu1—N1	172.63 (18)
C27—C18—C19—C20	−3.0 (4)	C6—N2—Cu1—N1	−10.71 (18)
C17—C18—C19—N3	−1.2 (3)	C24—N4—Cu1—N2	62.3 (2)
C27—C18—C19—N3	179.6 (2)	C21—N4—Cu1—N2	−110.21 (19)
N3—C19—C20—C21	1.0 (4)	C24—N4—Cu1—N3	173.7 (2)
C18—C19—C20—C21	−176.1 (2)	C21—N4—Cu1—N3	1.21 (19)
C19—C20—C21—N4	1.9 (4)	C24—N4—Cu1—N1	−54.6 (2)
C19—C20—C21—C22	−174.2 (2)	C21—N4—Cu1—N1	132.85 (18)
N4—C21—C22—C23	−0.6 (3)	C16—N3—Cu1—N2	−50.2 (2)
C20—C21—C22—C23	175.9 (2)	C19—N3—Cu1—N2	137.32 (18)
N4—C21—C22—C28	−179.6 (2)	C16—N3—Cu1—N4	173.8 (2)
C20—C21—C22—C28	−3.0 (4)	C19—N3—Cu1—N4	1.31 (19)
C21—C22—C23—C24	0.9 (3)	C16—N3—Cu1—N1	55.3 (2)
C28—C22—C23—C24	179.8 (2)	C19—N3—Cu1—N1	−117.27 (18)
C21—C22—C23—C29	−178.0 (2)	C1—N1—Cu1—N2	176.26 (19)
C28—C22—C23—C29	0.9 (4)	C4—N1—Cu1—N2	19.68 (18)
C22—C23—C24—N4	−0.9 (3)	C1—N1—Cu1—N4	−44.5 (2)
C29—C23—C24—N4	178.1 (2)	C4—N1—Cu1—N4	158.88 (16)
C22—C23—C24—C30	−179.3 (2)	C1—N1—Cu1—N3	67.6 (2)
C29—C23—C24—C30	−0.4 (4)	C4—N1—Cu1—N3	−89.01 (19)

### Hydrogen-bond geometry (Å, º)

Cg is the centroid of the N3/C16–C19 pyrrole ring.

**Table:**

*D*—H···*A*	*D*—H	H···*A*	*D*···*A*	*D*—H···*A*
C29—H29*C*···*Cg*^i^	0.98	2.78	3.551 (3)	136

Symmetry code: (i) *x*−1, *y*, *z*.
